# Concerted Collusion: Studying Multiagency Institutional Cover-Up

**DOI:** 10.3389/fpsyg.2022.847376

**Published:** 2022-06-16

**Authors:** Anthony Montgomery

**Affiliations:** Department of Psychology, Northumbria University Newcastle, Newcastle upon Tyne, United Kingdom

**Keywords:** cover-up, corruption, collusion and hierarchies, organizational ethical behavior, victims

## Abstract

Many important organizational events do not lend themselves easily to experimental manipulation, and thus, one can only study them retrospectively by combining the investigative tools provided by both the social sciences and humanities. A cover-up, meaning an attempt to prevent the public from discovering information about a serious crime or mistake, is such a phenomenon. The objective of the present paper is to develop an initial taxonomy of how organizational researchers can study what happens when multiple organizations and institutions conspire to cover-up the causes of a tragedy. For this purpose, the 1989 United Kingdom Hillsborough tragedy and the 27 year cover-up will be analyzed. Hillsborough is the best (and worst) example of a cover-up, in that the objective facts were known from early on but the subjective elements (i.e., attitudes, bias, and collusion) resulted in a 27 year search for justice for the victims. It deserves special attention as an example of multiagency institutional cover-up, in that the range and diversity of institutional actors pitted against the victims grossly outweighed them in terms of material resources, social power (in terms of social class differences), and the ability to control the narrative of the tragedy. Using a thematic analysis approach, five main themes were identified as: (1) Unwilling, but compliant, participants who are unlikely to be whistleblowers, (2) Suppressing/withholding important information, (3) Proactively engaging the support of related actors/institutions that helps create a critical mass, (4) Owning the narrative, and (5) Moral disengagement.

## Introduction

Many important organizational events do not lend themselves easily to experimental manipulation, and thus, one can only study them retrospectively by combining the investigative tools provided by both the social sciences and humanities. A cover-up, meaning an attempt to prevent the public from discovering information about a serious crime or mistake, is such a phenomenon. Arguably, institutional and organizational cover-ups are the most harmful and impactful elements of organizational life (Donziger, 1996). The impact of cover-ups includes, but is not limited to, the loss of trust in institutions, loss of legitimacy for guilty actors, additional pain and suffering for initial victims, and the loss of deserved financial redress. To date, the field has produced some excellent analyses of corruption at the organizational level which have included; Enron’s senior executives hiding the company’s precarious financial position (Eichenwald and Henriques, 2002), the United States Catholic Church covering up the predatory behaviors of pedophile priests (Terry and Smith, 2006), the Black Sox scandal whereby members of the Chicago White Sox Baseball team threw the 1919 World Series (Asinof, 1963), the Ford Pinto scandal (Gioia, 1992), and the Volkswagen emissions scandal (Hotten, 2015; Chossière et al., 2017). There is a considerable and comprehensive literature on the topic of organizational corruption (Anand et al., 1998, 2004; Ashforth et al., 2008); however, the specific phenomenon of cover-ups has been less systematically studied (Kundro and Nurmohamed, 2020). The present paper will specifically focus on the phenomenon of a wide scale cover-up, but it is important to note that there is overlap with the more generic field of organizational corruption. For example, Palmer (2008) notes that collective organizational wrongdoing involves the sustained coordination of multiple organizational participants.

The objective of the present paper is to develop an initial taxonomy of how organizational researchers can study what happens when multiple organizations and institutions conspire to cover-up the causes of a tragedy. For this purpose, the 1989 United Kingdom Hillsborough tragedy and the 27 year cover-up will be analyzed. Hillsborough is the best (and worst) example of a cover-up, in that the objective facts were known from early on but the subjective elements (i.e., attitudes, bias, and collusion) resulted in a 27 year search for justice for the victims. Moreover, unlike other examples of cover-ups, it involved concerted collusion among a large array of key societal actors, the police, senior politicians, journalists, healthcare professionals, and professional sports bodies. In this sense, it fits with the observation of Greve et al. (2010) that certain organizational contexts appear to generate misconduct, certain locations in networks appear to generate misconduct, and certain situations appear to generate misconduct. It deserves special attention as an example of multiagency institutional cover-up, in that the range and diversity of institutional actors pitted against the victims grossly outweighed them in terms of material resources, social power (in terms of social class differences), and the ability to control the narrative of the tragedy. Analyzing corruption and cover-ups at a wider societal level is in agreement with researchers who recommend that we look at corruption beyond individual organizations and make connections with the wider community (Ashforth and Gibbs, 1990; Getz, 2006).

Before analyzing Hillsborough in detail, the state-of-the-art in research on the phenomenon of cover-ups will be reviewed and the reader will be informed as to how the present analysis has built upon the work that has already been conducted.

## Research on Cover-Ups in Organizations

Research on the phenomenon of cover-ups has developed steadily over the last 50 years. For example, the Katz (1979) paper on concerted ignorance and the social construction of cover-up is a seminal paper. Katz, by his own admission, does not attempt to delineate the sources of cover-ups but rather he challenges the idea of employees as being deferential to strategic ignorance. Thus, there is an early recognition that cover-ups involved an active engagement by key stakeholders rather than simply the act of “turning a blind eye” to improper behavior. Early researchers also highlighted the importance of line managers in modeling unethical behaviors. In two consecutive surveys of *Harvard Business Review*, readers were asked to rank five factors according to their influence on unethical decisions (Baumhart, 1961; Brenner and Molander, 1977). Results indicated that the “behavior of superiors” was ranked as the most influential factor, followed by a cluster of factors—“formal policy or lack thereof,” “industry ethical climate,” and “behavior of one’s equals in the company”; tellingly, “one’s personal financial needs” came last. Congruently, the importance of leadership as a factor influencing corrupt behavior is evidenced by a review of corporate scandals among the Fortune 100 organizations, which concluded that the actions of the leaders (i.e., executives, boards of directors, and government officials) were primarily responsible for corruption (Clement, 2006).

Ashforth and Anand (2003) propose three pillars that contribute to the normalization of corruption in an organization: (1) institutionalization, the process by which corrupt practices are enacted as a matter of routine, often without conscious thought about their propriety; (2) rationalization, the process by which individuals who engage in corrupt acts use socially constructed accounts to legitimate the acts in their own eyes; and (3) socialization, the process by which newcomers are taught to perform and accept the corrupt practices. The importance of Ashforth’s and Annad’s paper is that they comprehensively address the question as to why an otherwise ethically sound person can become steeped in corruption? The highlight of the paper is the way the authors weave together different research strands concerning social influence and elucidate the way that socialization processes produce a self-fulfilling prophecy.

**Figure 1:**
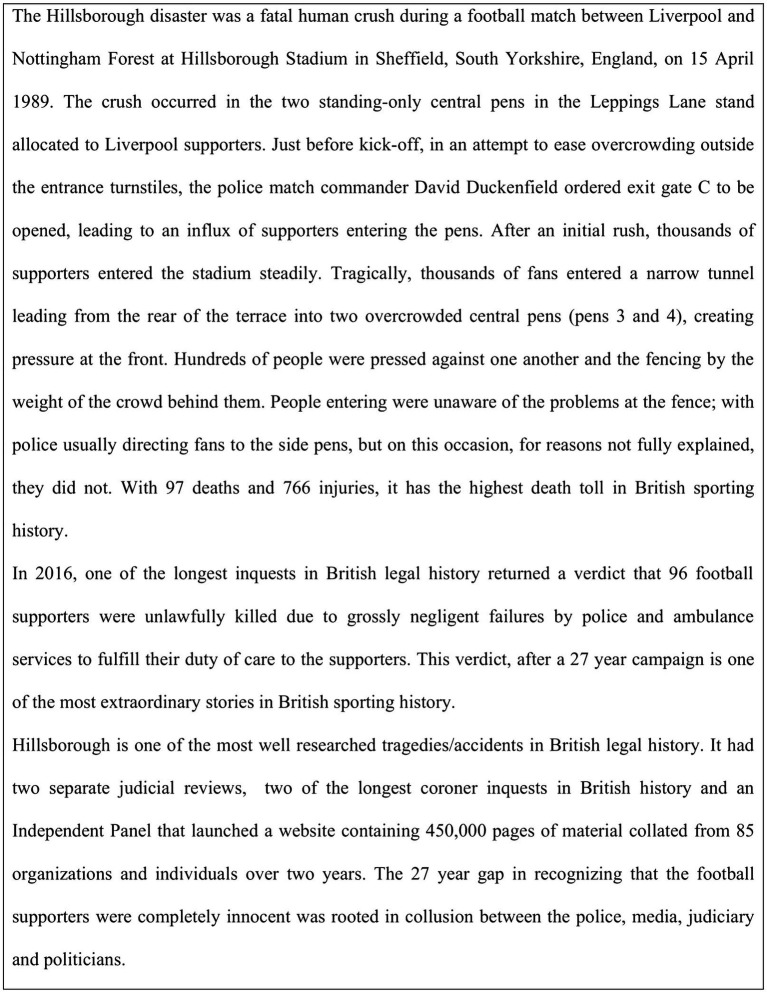
A brief history of the United Kingdom Hillsborough tragedy.

Overall, the accumulated work in this area has successfully provided organizational researchers with the mechanics of how corruption grows within an organization, is fostered, and becomes an inevitable outcome of a particular system (Treviño et al., 2014). However, organizations are embodied within neighborhoods, communities, cities, and a broader ethnic narrative. The present paper seeks to add to the field by developing a framework of how corruption is maintained beyond the “walls of an organization” and can become broader involving multiple stakeholders within a society. The tragedy of the United Kingdom Hillsborough disaster is the main focus of this paper (see Figure 1 for a brief history).

**Figure 2:**
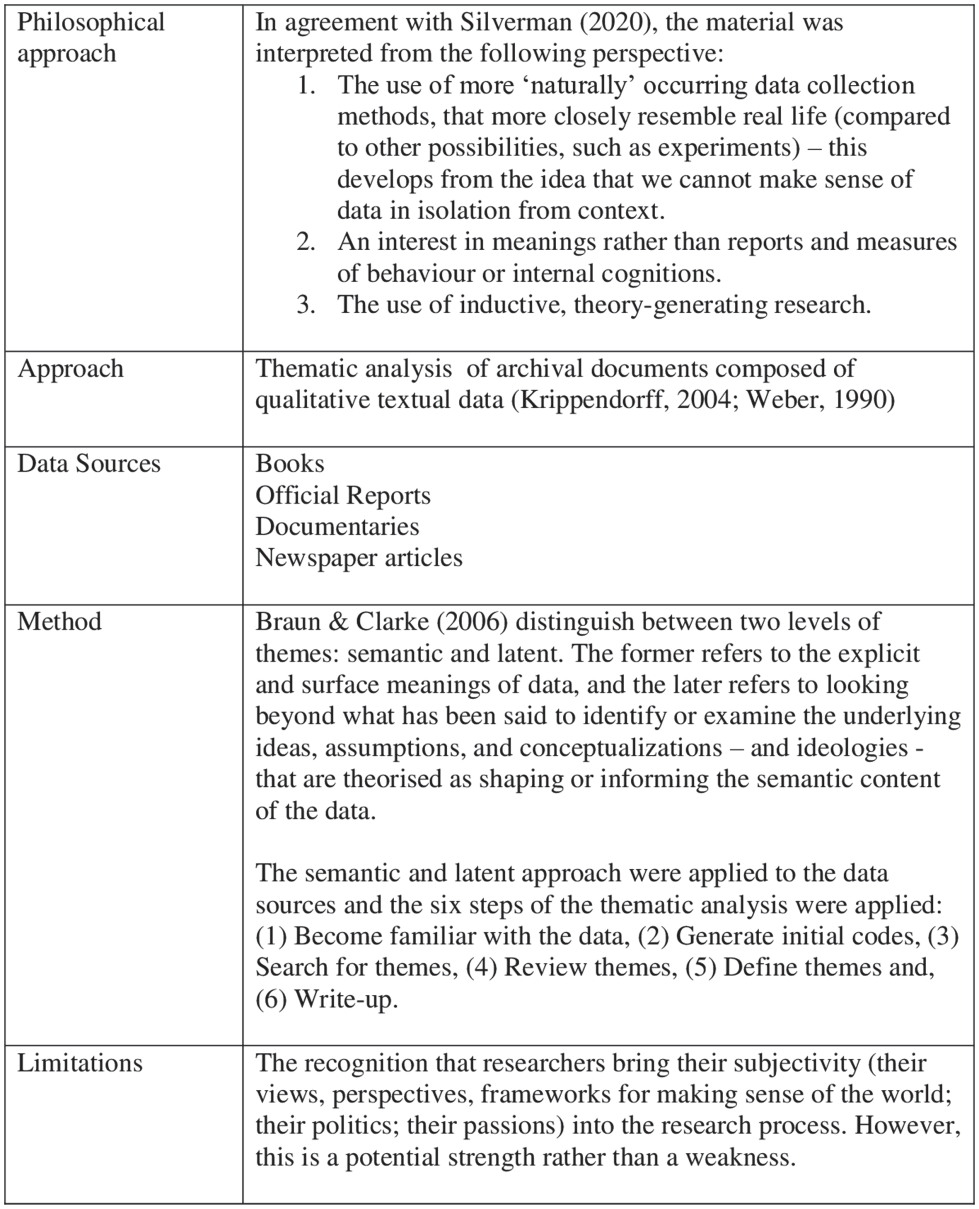
Thematic analysis of the Hillsborough disaster.

**Figure 3:**
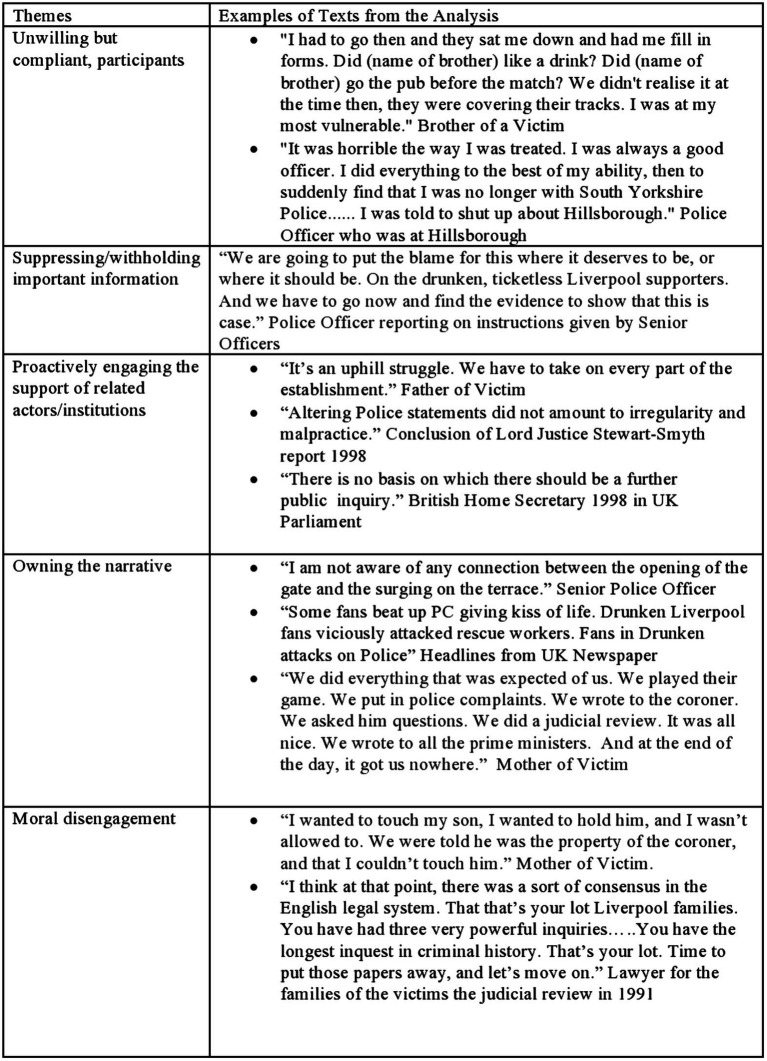
Thematic categories.

Hillsborough represents an unparalleled “natural” experiment in social and organizational psychology, in that this tragic event provides an opportunity to observe psychological theories and principles in retrospect. The analysis of historical events as a way to develop social-organizational theory has been used successfully (e.g., Haslam and Reicher, 2012; Stott et al., 2018). Using the large amount of information available on Hillsborough, I have utilized thematic analysis (Braun and Clarke, 2006) to create an initial taxonomy of the elements that are crucial for the development and maintenance of cover-ups (see Figures 2, 3 for more detail). The analysis identified five main themes: (1) Unwilling, but compliant, participants who are unlikely to be whistleblowers, (2) Suppressing/withholding important information, (3) Proactively engaging the support of related actors/institutions that helps create a critical mass, (4) Owning the narrative, and (5) Moral disengagement. In the following, I will describe each theme in detail.

## Unwilling, But Compliant Participants

One of the key elements in exacerbating the cover-up was the actions of the police force to knowingly edit witnesses’ statements to erase evidence of potential wrongdoing and bad decision making on the part of the police, such as suggesting that inappropriate behavior by football fans may have contributed to the tragedy. The challenge for the researcher is to understand how “ordinary” members of the force colluded with the highly skewed version of the events that emerged under the influence of more senior officers.

A number of television documentaries that sought to establish the real truth of Hillsborough presented interviews with low-ranking police officers who reported misgivings about the behavior of their organization both on the day of the tragedy and in the following years. The interviews with these morally distressed individuals also showed how reluctant they were to be “whistleblowers” and their fear of the retribution that their organizations might visit upon them. In this regard, the “Abilene paradox” is a useful explanatory mechanism (Harvey, 1974). The paradox involves a common breakdown of group communication in which each member mistakenly believes that their own preferences are counter to the group’s and, therefore, does not raise objections. The Abilene Paradox is a desire not to “rock the boat.”

In terms of understanding the behavior of the police, the concept of organizational memory is useful. For example, research on the dark side of policing highlights the norms that can support police corruption and which are integrated into organizational memory (Kappeler et al., 1994). Such norms include: not “ratting” on another officer, not implicating one’s colleagues if you are caught doing something, not interfering with the activities of other police officers, not trusting new people until they have been socialized into the norms, and not volunteering information about any event that could implicate a colleague. Not surprisingly, corrupt decisions that result in positive outcomes are included in organizational memory and provide guidelines for future behavior (Anand et al., 1998). Indeed, recent evidence suggests that “breaking the code of silence” in the police force is still rare and challenging (Lee and Helen, 2020).

Additionally, the literature on the Social Cocoon (Greil and Rudy, 1984) is useful in terms of understanding the behavior of the police and media concerning the tragedy. According to the concept of the social cocoon, the proximal forces (i.e., cognitions and emotions) are heavily influenced by distal ones (i.e., ongoing narrative and socialization practices of an organization). Thus, according to this analysis, the police and the media inhabit a psychologically encapsulated social cocoon where: (1) senior colleagues model the corrupt behavior and easy acceptance of it; (2) newcomers are encouraged to affiliate and bond with veterans; (3) low-ranking offices are subjected to strong and consistent information and ideological statements such that ambiguities and problematic actions are resolved in clear black and white terms (Janis, 1983); (4) low-ranking offices are reinforced to believe that their moral distress is a results of naivety; and (5) corrupt behaviors are valorized and doubt is projected as weakness/disloyalty (Brief et al., 2001). The social cocoon is most powerful when it fosters pluralistic ignorance among actors, meaning that police officers and journalists who are suffering from moral distress over the actions of their organizations believe that they are alone in their suffering (Janis, 1983).

## Suppressing/Withholding Important Information

Social psychological experiments have shown that individuals in the appropriate context can carry out extraordinary acts (Milgram, 1963; Zimbardo, 2007). This element seems particularly relevant regarding the behavior of the high-ranking police officers who established a subgroup of mid-ranking officers whose purpose was to alter the witnesses’ statements of low-ranking police officers; to erase/alter evidence that reflected badly on the police and include evidence that could impugn the reputations of the victims. Most recently, Kennedy and Anderson (2017) found that holding high-ranking positions makes people less likely to engage in principled dissent. According to the authors, high-ranking individuals identify more strongly with their organization or group, and therefore see its unethical practices as more ethical than low-ranking individuals do. The relationships between different actors in the police force can perpetuate shared norms of professional morality tolerant of serious misconduct, regardless of the harm caused to individuals or the organization (Hutchinson et al., 2009).

Ultimately, it is clear that the authorities (and the Police in particular) engaged in misbehavior. As noted by Argyris (1995), there is a paradox that behavior which we are taught leads to caring, support, honesty (etc.), also necessarily leads to lack of caring, distancing, and designed dishonesty and does so in ways that the latter consequences are covered up and the cover-up is covered up. The way in which the Police managed the amending of witnesses’ statements demonstrates this perfectly. Low-ranking police officers were provided with the opportunity to produce an open and free-flowing witness statement on blank paper, which was subsequently amended/edited (without their consent) for the final official version of the police witness statements. This practice also demonstrates the way that risk blindness (i.e., being unaware of the existence of organizational risks due to their lack of visibility) and safety drift (i.e., people deviating from, and/or failing to follow, policies, rules, regulations, and procedures; Goh et al., 2012) can lead to the paradoxical phenomenon that “a strong production focus can trigger a vicious cycle of deteriorating risk perception and how increased protection effort can, ironically, lead to deterioration of protection” (p. 69).

## Proactively Engaging the Support of Related Actors/Institutions That Helps Create a Critical Mass

One would have expected that journalists would be at the forefront of revealing injustice rather than colluding with the Police to smear the behavior of the victims. The narrative of the media was driven by a context in which football hooliganism was considered the norm in the United Kingdom in the 1980’s, and thus, initial explanations concerning the disaster suggested the behavior of football fans was most likely to blame. The discourse and vocabulary of corruption in the media helped to create, sustain, or challenge conceptions of organizational legitimacy, which means that it is an important arena for sensemaking and legitimation (Breit, 2010, 2011). The media created an “out-group” with their “hooligan” language that made it easier for the public to denigrate the victims. A wide range of newspapers published allegations that football fans had engaged in activities such as stealing from the dead, and assaulting police officers and rescue workers. The apotheosis of this idea reached its zenith when a popular British newspaper, *The Sun*, ran a front page story claiming that they had unearthed the “truth” about fan behavior at Hillsborough. The story alleged that Liverpool football fans had urinated on police officers, attacked police officers trying to give first aid to victims and stolen from dead victims. For the survivors of Hillsborough, this represented a second tragedy where they were blamed both for causing the crush at the football stadium and for hindering the efforts of the police to ameliorate the tragedy. Reporting in the aftermath of the tragedy incorrectly located the blame with the fans. Newspapers reported the following:

*Drunken Attacks on Police: Ticketless Thugs Staged Crush to Gain Entry* (Sheffield Star).

*Some fans picked pockets of victims. Some fans urinated on the brave cops* (Sun Newspaper).

*How long will it take for it publicly to be acknowledged that fans themselves share the blame?… The catastrophe was caused first and foremost by violent enthusiasm for soccer, in this case the tribal passions of Liverpool supporters. They literally killed themselves and others to be at the game* (Evening Standard).

The police reinforced their legitimacy *via* symbolic management (Ashforth and Gibbs, 1990). Symbolic management concerns the transformation of the meaning of acts (i.e., the victims behavior transformed into crimes against the police), and espousing socially acceptable goals while actually pursuing less acceptable ones. Such symbolic management leverages cross-institutional support and builds a critical mass.

## Owning the Narrative

The overriding public narrative was that the fan behavior was a significant cause of the tragedy, and successive inquires only compounded the picture of the survivors’ families as self-pitying, undeserving, and in denial about the behavior of their loved ones. Pinto et al. (2008), in their discussion of organizational corruption, make an important distinction between a corrupt organization (CO) and an organization of corrupt individuals (OCI). Their definition of a CO elucidates the processes that contributed to the institutional cover-up over Hillsborough. According to the authors, CO is usually a top-down phenomenon in which a group of organization members—typically, the dominant coalition, organizational elites, or top management team—undertake, directly or through their subordinates, collective and coordinated corrupt actions that primarily benefit the organization. Moreover, the authors further stipulate that it is an organization-level phenomenon since the organization is not only the primary beneficiary but also the primary entity culpable, even if the officers responsible are individually culpable as well. In essence, the OCI is a barrel full of bad apples, whereas the CO is the whole orchard. Hillsborough represents a coalition of forces against victims and their families that sought to bury the truth and aggressively resist the conclusion that key parts of British society had failed them. Research in organizational ethics and social identity suggests that in-group third parties may generally punish cover-ups less severely than out-group third parties (Tenbrunsel and Smith-Crowe, 2008; Hotten, 2015), and in-group third parties are more likely to justify the actions of an unethical group member and be willing to take their perspective (Galinsky and Moskowitz, 2000).

Finally, “owning the narrative” was concerned with how the key stakeholders (i.e., Police, Media, and Government agencies) constructed and controlled the sensemaking around Hillsborough. Fisher (1985) provides two concepts that are crucial to understanding both how the narrative is maintained and can be demystified. Narrative probability (i.e., the question of whether or not a story coheres or “hangs together”), and narrative fidelity (i.e., concerns the “truth qualities” of the story, and the soundness of its reasoning) were both features of how the false narrative continued, but also how the truth eventually emerged.

## Moral Disengagement

We normally expect that when the opportunity to engage in unethical behavior arises, our self-regulatory mechanisms (e.g., guilt and self-censure) should prevent us from engaging in such behavior. However, moral disengagement theory suggests that self-regulatory processes can be deactivated by the use of moral disengagement techniques, such as diffusing responsibility, displacing responsibility, blaming the victim, or claiming that the action is warranted because it serves a higher purpose (for a review, see Moore et al., 2012). As noted by Moberg (2006), people have a tendency to self-perceive themselves in terms of a competency framework rather than a moral framework. This means that the need to feel competent can be more important than the need to act morally, thus creating an ethical blind spot. So for example, when individuals assess their own attributes, they are much more prone to do so with terms like “clever, efficient, energetic, logical, and knowledgeable” than “fair, honest, loyal, sincere, and selfless” (Wojciszke, 2005). Consequently, the more that performance qualities (e.g., being clever) are part of our self-concept, the more likely we are to allow performance to override morally inappropriate actions (Sternberg, 2002).

Gradually, the real facts of Hillsborough started to emerge. This initially resulted in greater efforts by the various actors responsible for the tragedy to distance themselves from responsibility and reinforce the narrative of the football fans as behaving like a mob. Shaul Shalvi et al. (2015) have identified three psychological mechanisms people use as post violation justifications that are particularly relevant to Hillsborough: cleansing, confessing, and distancing.

*Cleansing* can take a symbolic or physical form. The cold and clinical treatment of the dead football fans and the families immediately after the tragedy represents perfectly the desire of the authorities to “cleanse” themselves of the responsibility. Some of the mothers of the victims reported that they were told that their deceased children were the property of the coroner and they were not allowed to hold them. Indeed, the treatment of the relatives immediately after the tragedy was akin to a criminal investigation with the victims as suspects rather than a compassionate process of communicating tragic news.

*Confessing* typically involves a partial confession. Thus, when they are genuinely regretful, people opt for partial rather than full confessions. Partial confessions allow people to feel moral for having the dignity to admit to some wrongdoing without having to bear the consequences of the full violation (Pe’er et al., 2014). The first major report (Taylor report) of the tragedy heavily criticized the police, but the report did not result in criminal prosecutions or the exoneration of the fans (Taylor, 1989).

*Distancing* means that when people cannot deny, confess, or compensate for their wrongdoings, they distance themselves from these transgressions, use stricter ethical criteria, and judge other people’s immoral behavior more harshly (Barkan et al., 2012). The systematic attempt to impugn the football fans as being drunk and abusive toward the police is a great example of distancing. Over the 27 years, there was a systematic and continuous attempts by some members of the police to malign the behavior of the football fans as a defense tactic (Scraton, 2009).

## Concluding Remarks

The story of Hillsborough fits with the arguments of Jagannathan and Rai (2017) that a sense of national crisis produced by national and organizational elites can alter ethical temporalities within broader levels of society and organizations such as the police, meaning that during a crisis, agents such as police officers find that they do not have enough dialogical time to raise ethical questions premised on moral anger. Consequently, climates of silence corruption perpetuate opportunities for institutions to marginalize, shun, and vilify those who “speak out” (Faunces and Bolsin, 2004).

There is significant lack of research concerning the phenomenon of society-wide cover-ups in the organizational literature. This paper is not exhaustive but has introduced an initial classification of elements crucial to maintaining a cover-up across different institutions. Future research needs to take an interdisciplinary approach to corruption (Breit, 2011) and explore how we can address corruption as a national narrative, which cannot be adequately addressed by the two most popular policy prescriptions for curbing collective organizational wrongdoing (i.e., governance reform and moral instruction). The challenge is to go beyond looking at just the factors that impinge on organizational participants and lead them to engage in wrongdoing in conjunction with others (Palmer, 2008), but to also identify the organizational, industrial, or higher level structural elements that are conducive to the growth of cover-ups.

## Data Availability Statement

The original contributions presented in the study are included in the article/supplementary material; further inquiries can be directed to the corresponding author.

## Author Contributions

The author confirms being the sole contributor of this work and has approved it for publication.

## Conflict of Interest

The author declares that the research was conducted in the absence of any commercial or financial relationships that could be construed as a potential conflict of interest.

## Publisher’s Note

All claims expressed in this article are solely those of the authors and do not necessarily represent those of their affiliated organizations, or those of the publisher, the editors and the reviewers. Any product that may be evaluated in this article, or claim that may be made by its manufacturer, is not guaranteed or endorsed by the publisher.
